# Endovascular Management of Cerebral Aneurysms of the Posterior Cerebral Artery

**DOI:** 10.3389/fneur.2021.700516

**Published:** 2021-10-21

**Authors:** Xin-Yu Li, Cong-Hui Li, Ji-Wei Wang, Jian-Feng Liu, Hui Li, Bu-Lang Gao

**Affiliations:** Department of Neurosurgery, The First Hospital, Hebei Medical University, Shijiazhuang, China

**Keywords:** posterior cerebral artery, aneurysm, endovascular embolization, stent-assisted, coiling

## Abstract

**Purpose:** To investigate the safety and efficacy of endovascular embolization of cerebral aneurysms at the P1–P3 segments of the posterior cerebral artery (PCA).

**Materials and Methods:** Seventy-seven patients with 77 PCA aneurysms who were treated with endovascular embolization were enrolled, including 35 (45.5%) patients with ruptured aneurysms and 42 (54.5%) with unruptured ones. The pretreatment clinical data and aneurysm occlusion status after treatment and at follow-up were analyzed.

**Results:** All patients were successfully treated endovascularly, including coiling alone in 10 (13.0%) patients, stent-assisted coiling in 18 (23.4%), parent artery occlusion in 25 (32.5%), and pipeline embolization device (PED) in 24 (31.2%). Complete occlusion was achieved in 48 (62.3%) aneurysms, residual neck in 4 (5.2%), and residual aneurysm in the other 25 (32.5%) at the end of embolization. Periprocedural complications occurred in eight patients, including acute thrombosis in seven (9.1%) and intraprocedural subarachnoid hemorrhage in one (1.3%), with the total complication rate of 10.4%. Follow-up was performed in 60 patients (77.9%) for 42 ± 11 months; the mRS score was 0–2 in 55 (91.7%) patients, three in four patients (6.7%), and six in one patient (1.7%). Fifty-three (88.3%) patients (53 aneurysms) had stable or complete occlusion, and seven (11.7%) patients had aneurysm recurrence or residual aneurysm. Among 19 patients treated with PED at follow-up, 15 aneurysms (78.9%) proceeded to complete occlusion while four (21.1%) aneurysms showed residual aneurysm.

**Conclusion:** Endovascular embolization remains a good choice of treatment with high safety and efficacy for posterior cerebral artery aneurysms.

## Introduction

Cerebral aneurysms at the posterior cerebral artery (PCA) are rare, accounting for <1% of all intracranial aneurysms and about 7% of the posterior circulation aneurysms (1–4). Few patients with PCA aneurysms have been reported in the literature because of the low incidence of aneurysms at this location (5–8), and the largest series of cerebral aneurysms at this location has been reported in 125 aneurysms, with the focus on the surgical treatment and outcome by Goehre et al. (1). PCA aneurysms are frequently multiple, and a relatively high proportion of the PCA aneurysms are nonsaccular, including fusiform, dissecting, or mycotic aneurysms (9). Fusiform aneurysms are classified as segmental ectasia with a stretched and fragmented internal elastic lamina, and dissecting aneurysms are those with widespread disruption of the elastic lamina, thickened intima, and extensive intraluminal thrombus (10). Most of the PCA aneurysms are located at the proximal P1 segment of PCA, with only 31% distal to the P1/P2 junction (1), and aneurysms beyond the P1/P2 junction are due to a dissection. The commonest location for saccular aneurysms is the P1 segment, and varied hemodynamic stresses at the proximal and distal segments of the PCA may play an important role in aneurysmal development at these sites. Open surgery for PCA aneurysms has a morbidity and a mortality as high as 13 and 19%, respectively (11), whereas endovascular embolization has become a major therapeutic approach for the PCA aneurysm because endovascular devices can be easily navigated to the location of aneurysm for treatment. However, most of PCA aneurysms are located at the P1 and P2 segments which are within the interpeduncular cistern adjacent to the brainstem and basilar artery tip harboring important perforating arteries, and proper indications and pretreatment evaluation are necessary for endovascular embolization so as to decrease the morbidity and mortality rates. In this study, we analyzed the endovascular management of PCA aneurysms and reported our results here. We used coiling embolization alone, stent-assisted coiling embolization, or Pipeline Embolization Device (PED, Medtronic, Dublin, Ireland) for treating these aneurysms.

## Materials and Methods

From February 2008 to October 2019, 77 patients with 77 aneurysms at the PCA were treated endovascularly in a single center, including 42 (54.5) male and 35 (45.5%) female patients with an age range of 18–76 (mean 53.2 ± 10.6) years (Table 1). The study was approved by the ethics committee of our hospital with the signed informed consent obtained from all patients or their family members. The clinical symptoms included subarachnoid or intraventricular hemorrhage from first rupture of cerebral aneurysms in 35 (45.5%) cases confirmed by computed tomography, with Hunt–Hess grade I in 8 (22.9%), II in 19 (54.3%), and III in 8 (22.9%) cases. Other symptoms included interval headache in 17 (22.1%) cases, oculomotor paralysis in 12 (15.6%), recurrent transient ischemic attack in 7 (9.1%), and blurred vision in 6 (7.8%). The aneurysms in four (5.2%) patients were incidentally found. Digital subtraction angiography demonstrated 14 aneurysms at the P1 segment (18.2%), 35 at P2 (45.5%), 17 at the P1–2 junction (22.1%), 6 at the P2–3 junction (7.8%), and 5 at P3 (6.5%). Saccular aneurysms were in 32 cases (41.6%), lobulated in 1 (1.3%), fusiform in 38 (49.4%), and dissecting in the remaining 6 (7.8%). The aneurysm was <5 mm in 14 (18.2%) cases, between 5 and 10 mm in 37 (48.15), 10–15 mm in 21 (27.3%), and > 15 mm in the remaining 5 (6.5%).

**Table 1:** Demographic features.

**Variables**		**Data**
No. of patients	77
F/M	35/42
Age (years, mean)	18–76 (53.2 ± 10.6)
Clinical presentations	Subarachnoid hemorrhage	35 (45.5%)
	HH grade I	8 (10.4%)
	HH grade II	19 (24.7%)
	HH grade III	8 (10.4%)
	Interval headache	17 (22.1%)
	Oculomotor paralysis	12 (15.6%)
	Transient ischemic attack	7 (9.1%)
	Blurred vision	6 (7.8%)
	Incidentally found	4 (5.2%)
Location of aneurysms	P1 segment	14 (18.2%)
	P2 segment	35 (45.5%)
	P1–2 junction	17 (22.1%)
	P2–3 junction	6 (7.8%)
	P3 segment	5 (6.5%)
Morphology of aneurysms	Saccular	32 (41.6%)
	Lobulated	1 (1.3%)
	Fusiform	38 (49.4%)
	Dissection	6 (7.8%)
Size of aneurysms	<5 mm	14 (18.2%)
	5–10 mm	37 (48.1%)
	10–15 mm	21 (27.3%)
	>15 mm	5 (6.5%)

Endovascular embolization was performed under general anesthesia with systematic heparinization: 3,000 units injected intravenously before the procedure and additional 1,000 units injected per hour until the end of the procedure. After transfemoral access, the guiding catheter was navigated to the vertebral artery. Coiling alone was performed for embolizing the aneurysm cavity of narrow-necked aneurysms, and stent-assisted coiling was conducted for wide-necked aneurysms. In case of giant and dissecting aneurysms, the parent artery was occluded with coils after occlusion test had proven to be endured by the patients. For stent-assisted coiling and PED, dual-antiplatelet therapy was administered. In case of hemorrhagic patients, tirofiban was injected intravenously at the dose of 10 μg/kg within 3 min at the time of stent deployment followed by intravenous infusion of tirofiban at the dose of 0.1 μg/kg/min for 24 h. After the procedure, dual-antiplatelet therapy was performed with clopidogrel 75 mg/day combined with aspirin 100 mg/day for at least 3 months. Clopidogrel response was evaluated with platelet aggregometry 3–5 days before the procedure, and patients with clopidogrel resistance were switched to prasugrel or ticagrelor.

Success of endovascular treatment was defined as successful deployment of endovascular devices with complete obliteration or partial occlusion of the aneurysm. Immediately following the procedure, angiography was performed to check for cerebral artery patency. Digital subtraction angiography was performed 3–6 months later. The Modified Rankin scale (mRS) was recorded at clinical follow-up after embolization. Aneurysm occlusion was evaluated with the Raymond–Roy classification system, with complete occlusion (100%) as class 1, residual neck as class 2, and residual aneurysm as class 3 (12, 13). Complications were evaluated, including possible intraprocedural bleeding and thrombosis, post-embolization neurological dysfunction, and new cerebral lesions on medical imaging.

### Statistical Analysis

The statistical analysis was performed with the SPSS 18.0 software package (IBM, Chicago, IL, USA). Continuous data were expressed as mean ± standard deviation, and the paired *t*-test was performed for statistical significance. The statistical significance was set at *p* < 0.05.

## Results

### Endovascular Treatment and Immediate Outcomes

All 77 patients were successfully treated endovascularly with a success rate of 100%, including parent artery occlusion in 25 patients (32.5%), coiling alone in 10 (13.0%), stent-assisted coiling in 18 (23.4%), and PED in 24 (31.1%; Table 1). No patients were transferred to open surgical clipping. Six patients with dissecting aneurysms and 19 with fusiform aneurysms underwent parent artery occlusion after they had been proven to be able to endure the occlusion test. Immediate angiography demonstrated complete occlusion in 48 (62.3%) aneurysms, residual neck in 4 (5.2%), and residual aneurysm in 25 (32.5%). One aneurysm with residual sac (class 3) was purposely occluded partially with stent-assisted coiling for avoiding occlusion of a perforating artery, and the other 24 aneurysms were treated with PED, resulting in class 3 occlusion with a residual sac.

### Periprocedural Complications

During the periprocedural period, seven (9.1%) patients had acute thrombus formation, leading to cerebral infarction in the posterior circulation in three patients (3.9%), but no sequelae in the other four patients after immediate thrombolysis. The cerebral infarction in the posterior circulation occurred in 3 (12%) of 25 patients with parent artery occlusion. One (1.3%) patient treated with stent-assisted coiling experienced intraprocedural subarachnoid hemorrhage. The total periprocedural complication rate was 10.4% (8/77). Among three patients with acute infarction, one patient had infarction in the cerebella confirmed by magnetic resonance image, resulting in diplopia and asynergy, and one had cerebellar lacunar infarction without apparent symptoms. The third one experienced large cerebral infarction after the ruptured aneurysm had been treated with parent artery occlusion, and follow-up after proper management showed left aphasia and right-side muscle weakness. The patient who had intraprocedural subarachnoid bleeding had no sequelae after proper medication.

### Follow-Up Outcomes

Follow-up was performed in 60 patients (77.9%) for 3 months to 7 years (mean 42 ± 11 months); the mRS score was 0–2 in 55 (91.7%) patients, 3 in 4 patients (6.7%), and 6 in 1 patient (1.7%). Fifty-three (88.3%) patients (53 aneurysms) had stable or complete occlusion, and seven patients exhibited aneurysm recurrence (in three patients or 5%) or class 3 occlusion (in four with PED or 6.7%; Table 2). The aneurysms in three patients were recurrent, including asymptomatic recurrence in one with initial parent artery occlusion, rebleeding in one with initial stent-assisted coiling, and asymptomatic recurrence in one with coiling alone. The recurrent aneurysm initially treated with stent-assisted coiling was retreated with stent-assisted coiling; however, re-rupture of the aneurysm 3 months later led to death of the patient (mRS 6). The recanalized aneurysm initially treated with parent artery occlusion was re-managed with parent artery occlusion, resulting in no clinical symptoms. A recurrent small aneurysm initially treated with coiling alone was recanalized due to coil compaction and remained in continual monitor because of the small size. Among 19 patients initially treated with PED, 15 aneurysms (78.9%) proceeded to complete occlusion while four aneurysms showed residual aneurysm (class 3 occlusion) at follow-up. No delayed complications occurred in patients who were treated with PED.

**Table 2:** Aneurysm occlusion and complications of endovascular treatment.

**Therapy**	**No. (%)**	**Immediate occlusion**			**FU occlusion**			**Complications**	
		**CO**	**RN**	**RA**	**CO**	**REC**	**RA**	**PEC**	**FU**
PAO	25 (32.5%)	25	0	0	19	1	0	3	0
CA	10 (13.0%)	7	3	0	7	1	0	1	0
SAC	18 (23.4%)	16	1	1	12	1	0	2	1
PED	24 (31.1%)	0	0	24	15	0	4	2	0
Total	77 (100%)	48	4	25	53	3	4	8	1

*PAO, parent artery occlusion; CA, coiling alone; SAC, stent-assisted coiling; P pipeline embolization device; CO, complete occlusion; RN, residual neck; REC, recurrence; RA, residual aneurysm; PEC, periprocedural; FU, follow-up*.

## Discussion

### Major Findings

In this study investigating the safety and efficacy of endovascular embolization of 77 cerebral aneurysms at the P1–P3 segments of PCA in 77 patients, it was found that endovascular embolization remained a good choice of treatment of PCA aneurysms with high safety and efficacy.

### Definition of PCA Segmentation

Currently, the definition of PCA segment is based on surgical data (1, 14). Anatomically, the PCA is divided into four segments. The P1 segment extends from the basilar artery tip to the posterior communicating artery (Pcom) origin. The cerebral peduncle constitutes the medial border of the P1 segment, and the occulomotor nerve courses between the P1 and the proximal superior cerebellar artery. The P2 segment extends from the Pcom to the midbrain dorsal aspect. From the P1 and P2 segments, perforating arteries to the thalamus and brain stem arise. The P3 and P4 segments extend distally from the midbrain dorsal aspect and supply the calcarine and parieto-occipital cortex.

### Characteristics of PCA and Parent Artery Occlusion

In the middle and anterior cerebral arteries, the incidence of fusiform aneurysms is only about 1%; however, the PCA has an exceptionally high incidence of fusiform aneurysms ranging from 19% up to 40% reported in the literature (1, 5, 7, 15). In our series, the fusiform aneurysm accounted for 49.4% (38/77). The PCA segment has lower blood flow but a higher incidence of fusiform aneurysm, which is probably caused by wall shear stress of blood flow damaging the vascular intima or direct injury to the PCA by the edge of the cerebellar tentorium because the P2 segment is closer to the tentorial edge and is the most affected segment by fusiform aneurysms (16, 17). Because most PCA aneurysms are fusiform or dissecting aneurysms with pathological damage of the PCA, occlusion of the parent artery is still one of the most applied approaches in treating the PCA aneurysms. The theoretical bases for parent artery occlusion lie in the fact that the PCA harbors abundant collateral circulation to compensate for parent artery occlusion, including anastomosis between perforating arteries of the brainstem (5, 14, 16). In our study, almost one-third PCA aneurysms were treated with parent artery occlusion (32.5%), resulting in only three acute posterior circulation infarctions (3.9%), including one disability (1.3%). Moreover, only one aneurysm was recurrent without any symptoms during follow-up, demonstrating the safety and efficiency of parent artery occlusion for PCA aneurysms, which is consistent with another report (5).

### Ischemic Complications and Possible Prevention Measures

In our study, one large cerebral infarction occurred, resulting in severe disability of the patient. This patient had concurrent occlusion of the ipsilateral middle cerebral artery, and the PCA was markedly enlarged to provide blood supply to the anterior circulation. Because the fusiform aneurysm in this patient was dissecting, occlusion of the parent artery was the best approach to decrease the hemorrhage risk; however, occlusion of the parent PCA caused severe ischemia to the ipsilateral brain and consequently large infarction. If an artery bypass was performed before occlusion, the ischemia could be avoided. In another case with a new infarction, the fusiform aneurysm involved most of the P1 segment, and occlusion of the parent artery might lead to low perfusion or even infarction in the distal portions of the brain. The high-risk factors for ischemic complications in parent artery occlusion are summarized as follows: poor collateral circulation especially with concurrent severe arterial stenosis or occlusion in the ipsilateral internal carotid artery system, enlarged parent artery with increased blood supply to the brain, and involvement of a longer segment of the parent artery. In managing patients with these risk factors, poor collateral circulation and thrombosis development affecting the basilar artery tip are relative contraindications, which can be resolved by use of endovascular means or artery bypass to improve the collateral circulation and perfusion before performing parent artery occlusion. Treatment of these patients can begin by embolizing the aneurysm dome and later occluding the parent artery so as not to affect the normal artery segment, especially the P1 and P2 segments. Moreover, sufficient use of antithrombotic medications can also reduce complications. Others suggest occlusion only of the parent artery without packing the aneurysm cavity especially for giant or extremely lobulated aneurysms because parent artery occlusion alone can lead to decreased pressure within the aneurysm sac, which will later collapse without causing an occupying effect (18). However, this embolizing approach has been reported to have a high short-term recurrence rate and a high morbidity after hemorrhage caused by recurrence (19). In our study, we used a strategy of occluding both the aneurysm cavity and the parent artery at the same time without increasing complications caused by the occupying effect of aneurysm packing.

### Stent-Assisted Coiling

As a reconstructive rather than deconstructive approach, stent-assisted coiling has become the first choice for managing most cerebral aneurysms, especially at the P1 and P2 segments, because it can keep the parent artery unobstructed without affecting small perforating arteries (19, 20). However, this type of treatment may have higher recurrence and bleeding rates in treating PCA aneurysms (21–23). In our study, the only bleeding complication occurred in one of the patients treated with stent-assisted coiling. PCA aneurysms are mostly large or giant dissecting aneurysms with lobulated morphology which cannot be embolized evenly and densely in stent-assisted coiling, leading to possible recanalization. Hemodynamic changes caused by embolization may result in fatal bleeding because of acute thrombosis within the aneurysm cavity and ischemia in the aneurysm wall even though medical imaging has demonstrated dense packing of the aneurysm. Antithrombotic treatment following endovascular management may also lead to uncontrollable hemorrhage. Moreover, due to the small vessel diameter of the P1 and P2 segments, collapse of the stent may take place and cause in-stent stenosis and occlusion in the long run (24).

### Coiling Alone

Coiling alone is the traditional method for endovascular embolization of cerebral aneurysms. However, in our study, we used this method only in 10 patients with small narrow-necked aneurysms because these aneurysms were saccular rather than fusiform or dissecting, and coiling alone had a lower risk of hemorrhage. In one case of unruptured aneurysm, coiling alone was performed to class 3 occlusion (residual aneurysm) of the aneurysm because the parent artery was very small and unsuitable for stenting while the collateral circulation might not endure parent artery occlusion. Partial embolization of the aneurysm sac plus close follow-up may be the best approach for this patient.

### Flow Diversion

Flow-diverting devices are a type of stent with metal coverage as high as 30% which emerged over 10 years ago. They work through alteration of the hemodynamics, leading to thrombosis within the aneurysm cavity, especially for large and giant aneurysms in the anterior circulation. Initial experience with the flow-diverting devices was mixed with poor outcomes (25, 26), especially in the posterior circulation. The flow-diverting devices have a higher complication rate in the posterior than in the anterior circulation (16 vs. 8%) (27). However, with experience accumulation, increasingly good outcomes of treatment for the PCA fusiform aneurysms with the flow-diverting devices have been reported (28, 29). Bhogal et al. (28) presented the largest series of 56 patients with nonsaccular aneurysms including 24 fusiform ones in the PCA, with the mortality rate of 4% (one death), complete occlusion of 75%, and minor residual filling in 12.5%. Zumofen et al. (30) reported good outcomes in applying the PED in treating six nonsaccular aneurysms of the proximal PCA, resulting in patency of the parent PCA but no new permanent neurological sequelae. In our study, the flow-diverting devices were used in 24 patients, resulting in class 3 occlusion (residual aneurysm) immediately after the procedure in all 24 (100%) aneurysms, and 15 (62.5%) aneurysms proceeded to complete occlusion at follow-up. No delayed complications (thrombosis or hemorrhage) occurred at follow-up in this cohort.

### Study Limitations

Some limitations existed in this study including no control group of patients with aneurysms at other segments, being a single-center study, and incomplete angiographic follow-up in the patients, which may affect the generalization of the outcomes of this study. Future studies will have to address these problems for better efficacy.

## Conclusion

In conclusion, endovascular embolization remains a good choice of treatment with high safety and efficacy for posterior cerebral artery aneurysms.

## Data Availability Statement

The raw data supporting the conclusions of this article will be made available by the authors, without undue reservation.

## Ethics Statement

The studies involving human participants were reviewed and approved by Committee of The First Hospital of Hebei Medical University. The patients/participants provided their written informed consent to participate in this study.

## Author Contributions

C-HL: study design. X-YL, J-WW, J-FL, and HL: data collection. C-HL and B-LG: data analysis. X-YL: writing of the original version. B-LG: revision. HL: supervision. All authors approved the paper.

## Conflict of Interest

The authors declare that the research was conducted in the absence of any commercial or financial relationships that could be construed as a potential conflict of interest.

## Publisher's Note

All claims expressed in this article are solely those of the authors and do not necessarily represent those of their affiliated organizations, or those of the publisher, the editors and the reviewers. Any product that may be evaluated in this article, or claim that may be made by its manufacturer, is not guaranteed or endorsed by the publisher.
